# Memory-like innate lymphoid cells in the pathogenesis of asthma

**DOI:** 10.3389/fimmu.2022.1005517

**Published:** 2022-11-17

**Authors:** Jongho Ham, MinYeong Lim, Dongmo Kim, Hye Young Kim

**Affiliations:** ^1^ Department of Biomedical Sciences, Laboratory of Mucosal Immunology, Seoul National University College of Medicine, Seoul, South Korea; ^2^ Department of Biomedical Sciences, BK21 Plus Biomedical Science Project, Seoul National University College of Medicine, Seoul, South Korea; ^3^ CIRNO, Sungkyunkwan University, Suwon, South Korea; ^4^ Institute of Allergy and Clinical Immunology, Seoul National University Medical Research Center, Seoul, South Korea

**Keywords:** innate lymphoid cell, memory, epigenetic change, asthma, trained immunity

## Abstract

Innate lymphoid cells (ILCs) are recently discovered innate immune cells that reside and self-renew in mucosal tissues and serve as the first line of defense against various external insults. They include natural killer (NK) cells, ILC1s, ILC2s, ILC3s, and lymphoid tissue inducer cells. The development and functions of ILC1–3 reflect those of their adaptive immunity T_H_1, T_H_2, and T_H_17 T-cell counterparts. Asthma is a heterogeneous disease caused by repeated exposure to specific allergens or host/environmental factors (e.g., obesity) that stimulate pathogenic pulmonary immune cells, including ILCs. Memory used to be a hallmark of adaptive immune cells until recent studies of monocytes, macrophages, and NK cells showed that innate immune cells can also exhibit greater responses to re-stimulation and that these more responsive cells can be long-lived. Besides, a series of studies suggest that the tissue-resident innate lymphoid cells have memory-like phenotypes, such as increased cytokine productions or epigenetic modifications following repetitive exposure to allergens. Notably, both clinical and mouse studies of asthma show that various allergens can generate memory-like features in ILC2s. Here, we discuss the biology of ILCs, their roles in asthma pathogenesis, and the evidence supporting ILC memory. We also show evidence suggesting memory ILCs could help drive the phenotypic heterogeneity in asthma. Thus, further research on memory ILCs may be fruitful in terms of developing new therapies for asthma.

## Introduction

Asthma is classically considered a chronic lung disease resulting from allergic responses characterized by airway obstruction and excessive mucus production. It is one of the most prevalent chronic lung diseases globally: more than 300 million people have asthma currently (1). Asthma was long thought to be an allergic disease that is mediated by excessive T-helper (T_H_)2-cell production of interleukin (IL)-4, IL-5, IL-9, and IL-13, which drive immunoglobulin E production, eosinophilia, mast-cell activation, and mucus secretion by airway epithelial cells (2). However, asthma is a complex disease that is now known to comprise another endotype, namely, non-allergic asthma: 10-33% of asthmatics have non-allergic asthma triggered by obesity, ozone exposure, or air pollutants (3). For example, 5–10% of asthmatics have severe asthma resistant to inhaled corticosteroids, which is the cornerstone of asthma treatment; these patients consequently account for much of asthma-related morbidity and mortality. Further indicating the complexity of asthma, two-thirds of severe-asthma cases are characterized by neutrophilia rather than eosinophilia (4, 5).

The fact that steroids mainly target adaptive immune cells such as T and B cells suggests that the severe asthma phenotype may involve other immune cells (6). This observation has galvanized research into the roles of cells other than adaptive immune cells in asthma. Of particular interest may be innate lymphoid cells (ILCs). These are innate immune cells that mostly reside and self-renew in mucosal tissues (although they can also be found in the blood) and are now understood to play essential and occasionally pathogenic roles in the mucosae, including the lung. They have to date been divided into five types, namely, natural killer (NK) cells, ILC1s, ILC2s, ILC3s, and lymphoid tissue inducer (LTi) cells (7). The possibility that ILCs contribute to asthma is supported by many studies showing ILC2 frequencies are elevated in the periphery and lung of asthmatics (8). Moreover, there is growing evidence that ILC1 and ILC3 cells also participate in different asthma phenotypes, such as neutrophil-dominated asthma (9). In addition, murine models of acute onset and chronic asthma clearly show that ILCs are involved in various stages of asthma (10).

Since ILCs were first reported in the last decade, research on these cells lags behind that on adaptive immune cells. Interestingly, however, the functions (cytokine production) of ILC1s, ILC2s, and ILC3s have been found to mirror those of the adaptive CD4^+^ T-cell subsets known as T_H_1, T_H_2, and T_H_17 cells, respectively (11). Specifically, ILC1s produce interferon (IFN)-γ, transforming growth factor (TGF)-β, and tumor necrosis factor (TNF)-α; ILC2s secrete IL-4, IL-5, and IL-13; and ILC3s generate IL-22 and IL-17. However, unlike T cells, ILCs lack antigen-specific receptors and thus cannot be activated by specific antigens. Instead, they respond directly to local environmental signals produced during tissue damage (7, 11). For example, asthma associates with damaged epithelial cells and activated myeloid cells, which produce alarmins such as IL-15, IL-25, thymic stromal lymphopoietin (TSLP), and IL-33, have been observed to directly activate ILC2s (12, 13).

Immunological memory is characterized by immune cell reprogramming that causes the cell to become long-lived and respond much faster and more strongly to its triggering stimulus (14, 15). Until recently, it was a hallmark of adaptive immunity that distinguished it from innate immunity (16). However, increasing studies on particularly monocytes, macrophages, and NK cells suggest that innate immune cells can also develop memory (17, 18). Furthermore, emerging studies suggested that ILCs can acquire memory phenotypes in the context of repeated allergen challenges or infection (19, 20). In this review, we will discuss ILC biology, its roles in asthma, and the emerging evidence that suggests the memory characteristics of ILCs. Finally, we will discuss the possibility that memory ILCs may play an essential role in the heterogeneity of asthma.

## Biology of innate lympoid cells

ILCs differentiate in the fetal liver and adult bone marrow (21–24). ILC differentiation in the bone marrow is well-defined, particularly in the mouse. Thus, like T cells, the five ILC types arise from the common lymphoid progenitor (CLP). The ILC differentiation process then involves three sequential ILC progenitor cells, namely, early ILC progenitors (EILPs; these are the earliest common ILC precursors), common helper ILC precursors (CHILPs), and ILC precursors (ILCPs). These differentiation steps are regulated by the coordinated expression of transcription factors that activate or repress critical target genes. In the mouse, these include *Id2*, *Nfil3*, *Zbtb16* (which encodes PLZF), *Tcf7*, and *Gata3* (7, 25). *Nfil3* plays a particularly important role since it orchestrates the differentiation of all ILCs (26–28).

The EILPs consist of specified EILPs, which retain dendritic cell (DC) potential, and committed EILPs that have lost their DC potential and are committed ILC progenitors (29, 30). The committed EILPs give rise to either NK cell progenitors or CHILPs. The CHILPs, in turn, develop into either LTi cells or ILCPs. When the latter express PLZF, they turn into ILC1s, ILC2 progenitors (ILC2P), and ILC3s (31–34). ILC2Ps are functionally immature compared to lung ILC2s but can differentiate efficiently into ILC2s in mucosal tissues when transplanted into lymphoid-deficient mice (35).

NK cells bear remarkable cytotoxic capacity and also produce interferon (IFN)-γ and perforin. Consequently, they play key roles in anti-viral and anti-tumor immune responses. Similarly, by secreting large amounts of IFN-γ and cytotoxic molecules such as granzyme C in the liver and salivary glands, ILC1s also participate in defending the host from endogenous pathogens (36, 37). By contrast, ILC2s produce IL-5, IL-13, and amphiregulin, thereby helping clear parasites. Moreover, they can initiate allergic immune responses (38, 39). While ILC3s and LTi cells develop from different precursor cells (ILCPs and CHILPs, respectively), both secrete IL-17A and IL-22 and control extracellular microbes in mucosal tissues (40). However, LTi cells arise earlier during embryogenesis and participate in secondary lymphoid organogenesis, T cell tolerance, and T and B cell functions (41).

After they develop in the fetal liver or bone marrow, ILCs migrate to other tissues, including the lymph nodes, secondary lymphoid tissues such as Peyer’s patches, and the mucosae (42). In the mucosa, they undergo self-renewal in response to various environmental and host stimulants, both homeostatic and uncontrolled inflammatory conditions (22, 43).

## Innate lympoid cells in asthma

**Figure 1:**
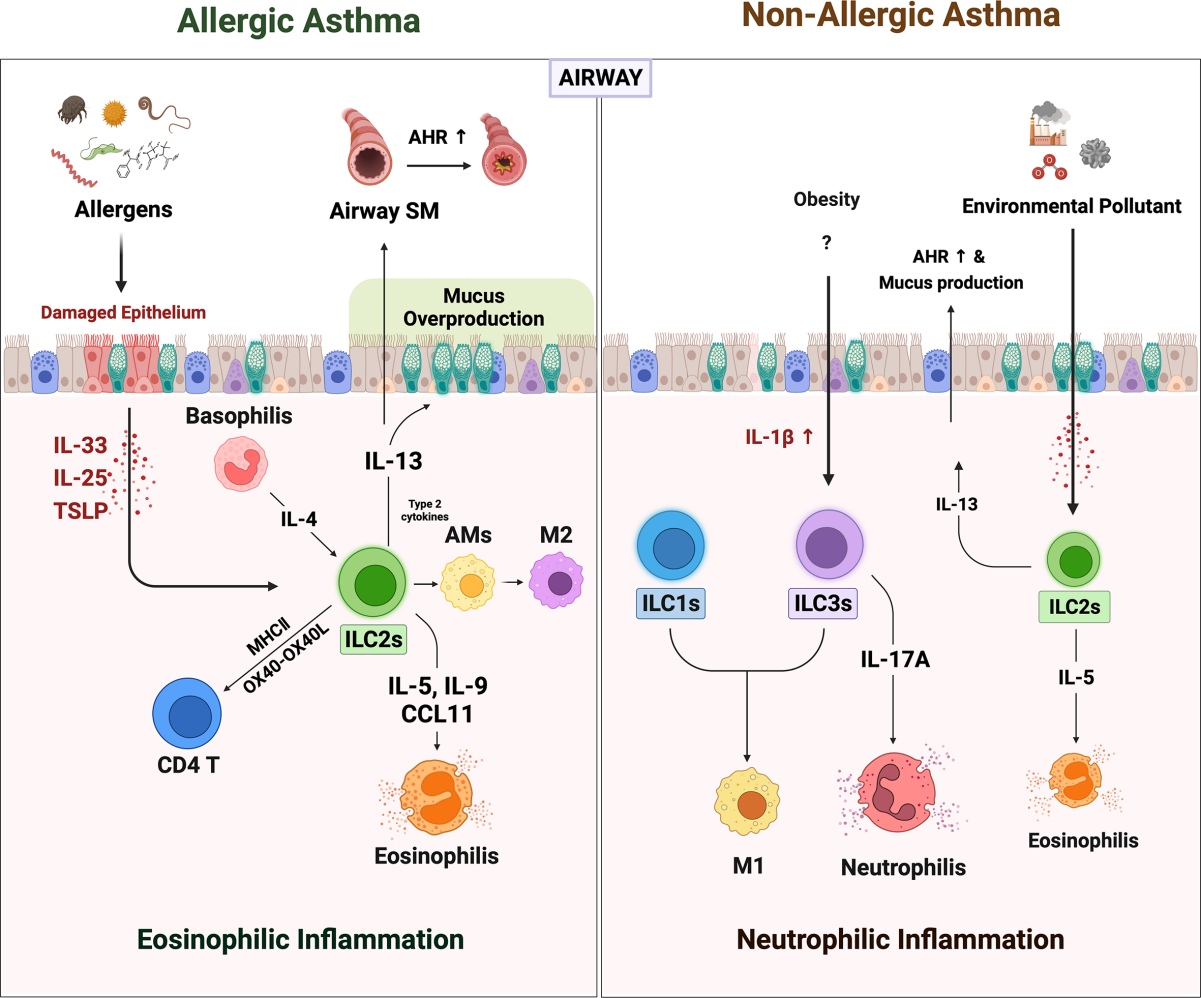
The role of ILCs in allergic asthma and non-allergic asthma. When the allergens irritated the airway epithelium, they secreted alarmins such as IL-25, IL-33, and TSLP. ILC2s are activated by alarmins and IL-4 from basophils. Activated ILC2s secreted large amounts of IL-5, 9, and CCL11, which derived recruitment of eosinophils. The ILC2s-derived IL-13 acts on airway epithelium and airway smooth muscle cells to induce mucus overproduction and airway hyperresponsiveness (AHR), respectively. Also, ILC2s-derived type 2 cytokines lead to the polarization of alveolar macrophages (AMs) to alternately activated M2-like phenotypes. ILC2s can directly activate CD4^+^ T by OX40-OX40L interaction or antigen presentation through MHCII molecules. Non-allergic asthma presents a more heterogeneous phenotype with severe neutrophilic inflammation than eosinophilia. In non-allergic asthma induced by obesity and air pollution, the number of ILC1s and ILC3s besides ILC2s is increased. Increased IL-1β in obese lungs not only induces neutrophilia by promoting IL-17 production from ILC3s but also increases pulmonary inflammation by leading to M1 polarization (which also contributes to ILC1 in this case). In addition, the increased numbers of the ILC2s in obesity and air pollution-induced asthma may synergistically exacerbate asthma.

A seminal study on virus-infected murine lungs was the first to identify ILCs, which were later classified as ILC2s (44). Further research on this cell led to a breakthrough finding in the asthma field. This means that, like T_H_2 cells, ILC2s can play key roles in asthma pathogenesis (45, 46). Of particular interest was the fact that ILC2s can both initiate and regulate the immune responses in asthma because they produce effector cytokines as well as innate immune cells. This was found later to be a general feature for ILCs since ILC1s and ILC3s play similar roles in non-allergic asthma (**Figure 1**). The evidence supporting these roles of ILCs in asthma will be reviewed below.

### Innate lymphoid cells in allergic asthma

Allergic asthma is the most common type of asthma. It is triggered by inhaled foreign allergens such as house dust mites (HDM), pet dander, pollen, and mold. Numerous studies show that these environmental insults irritate the airway epithelial cells and cause them to promptly increase their secretion of the alarmin cytokines, such as IL-33, IL-25, and TSLP. The importance of these alarmins in allergic asthma is demonstrated by a recent genome-wide association study (GWAS), which showed that genetic polymorphism in the gene encoding IL-33 or its receptor IL-1RL1 (ST-2) associates closely with the development of allergic asthma (47, 48).

Since IL-33 (and the other epithelial alarmins IL-25 and TSLP) is a major activator of ILC2s, the GWAS finding suggested that allergen-induced IL-33 may trigger local ILC2s. Indeed, it was shown that the relationship between IL-33 and ILC2s directly shapes the clinical symptoms of allergic asthma. Thus, several studies reported that both the IL-33 levels and ILC2 frequencies in the peripheral blood (49) and lung lavage fluid (50) of asthma patients correlate negatively with lung function as well as with each other. Moreover, a series of animal studies showed together that the epithelial alarmin-ILC2 relationship drives the eosinophil infiltration, airway hyperresponsiveness (AHR), and mucus production that characterizes allergic asthma, and that this effect does not depend on adaptive immune cells, as follows (51–54). First, when recombination-activating gene (*Rag*)-deficient mice (which bear ILCs but lack T and B cells) were subjected to intratracheal IL-33 and IL-25 administration, they developed eosinophilic airway inflammation (51). However, this was not observed in the *Rag* and *Il2rg* deficient mice that lacked all lymphocytes, including ILCs (53). Thus, alarmin administration by itself induces asthma *via* ILCs. Second, known asthma allergens, including fungal extracts, HDM, cockroaches, and pollens, trigger airway epithelial-cell secretion of alarmins. This effect is mediated by the destructive protease activities of the allergens (54). Several studies show that this process also involves ILCs. Thus, intratracheal administration of HDM or papain induces IL-5-producing ILC2s in the lungs along with airway inflammation, eosinophil infiltration, and mucus overproduction in mice. The latter effects are not observed when ILC2s are absent (52, 55). Moreover, treatment of naïve lung explants with papain induces the airway epithelial cells to secrete IL-33 and the ILC2s to produce IL-5 and IL-13 (52). Similarly, airway exposure to the common fungal aeroallergen *Alternaria alternata* upregulates IL-33, IL-5, and IL-13 levels in the bronchoalveolar lavage of mice and induces eosinophilic airway inflammation, however, this inflammation does not develop when the mice lack IL-33 or ILC2s (56, 57). Thus, allergens elevate epithelial-cell secretion of alarmins, which activate ILC2s in the lung, which in turn leads to the pathological effects of allergic asthma, namely, eosinophil infiltration, AHR, and mucus overproduction (58–61).

ILC2s also induce these pathological effects by promoting the polarization of alveolar macrophages in the lungs towards the alternatively activated M2 phenotype, which is known to induce eosinophilia (62). It is shown by the fact that co-culture of alveolar macrophages with ILC2s increases their expression of M2 signature genes, and eosinophilic asthma patients bear more ILC2s and M2 macrophages in their induced sputum than healthy controls (8). ILC2s also promote allergic asthma by facilitating the asthmogenic role of memory T_H_2 cells. This is shown by a study with an HDM/papain-induced murine model of asthma that requires antigen sensitization before the challenge: it was observed that when lethally radiated mice were transplanted with bone marrow from ILC2-deficient mice then sensitized and challenged with HDM/papain, their airway inflammation was strongly attenuated. It was accompanied by a significantly weaker T_H_2 response to the antigen. It was thought that this reflected poor T_H_2 priming due to the lack of IL-13-induced migration of activated lung DCs to the draining lymph node (60). Similarly, another study suggested that ILC2s shape memory T_H_2 cell activity by promoting local DC release of CCL17, which is essential for memory T_H_2 cell infiltration (63). *In vitro* studies show that ILC2s can also directly upregulate T_H_2 cell responses by binding to the OX40 ligand on the T cells (64) and/or by expressing MHC class II (65). Together, these observations demonstrate the crucial importance of ILC2s in allergic asthma. Finally, it should be noted that the roles of ILC2s in allergic asthma can be regulated by other innate immune cells. Thus, Motomura et al. showed that ILC2-induced eosinophilic inflammation can be augmented by basophils, whose secretion of IL-4 increases ILC2 expression of CCL11 (an eosinophil chemoattractant), IL-9, and IL-13 (66). Conversely, mast cells downregulate IL-33- or papain-induced eosinophilic inflammation by producing IL-2, which expands regulatory T cell numbers, thereby increasing their suppression of ILC2s (51). Further studies on the interactions between ILC2s and other innate immune cells are likely to reveal more such up- and down-regulatory interactions.

### Innate lymphoid cells in non-allergic asthma

Up to a third of asthmatics have forms of asthma whose triggers are not allergens. Moreover, these phenotypes are often dominated by neutrophils rather than eosinophils, which supports the notion that the pathogenic mechanisms in non-allergic asthma differ from those of allergic asthma. One such phenotype is obesity-induced asthma (67). This phenotype seems to be driven by the upregulation of IL-1β in the obese lung, which in turn potently stimulates ILC3s in the lung (68, 69). Kim et al. showed that when mice became obese due to a high-fat diet, they spontaneously developed AHR accompanied by significantly increased pulmonary numbers of IL-17A-expressing CCR6^+^ ILC3s. The activation of ILC3s in the obese mice was mediated by NLR family Pyrin domain-containing (NLRP)3 inflammasome-derived IL-1β in classically activated (M1) macrophages: NLRP3 deletion or treatment with anakinra (IL-1Ra) attenuated obesity-induced asthma. Moreover, intratracheal administration of IL-1β induced AHR by increasing pulmonary IL-17A levels. Notably, the role of ILC3s in this asthma phenotype was independent of adaptive immune cells since *Rag^−/−^* mice fed a high-fat diet developed AHR unless *Il2rg* was also deleted. Moreover, adoptive transfer of IL-17-producing ILC3s into *Rag2^−/−^Il2rg^−/−^* mice restored IL-1β-induced AHR. Thus, IL-17-producing ILC3s were necessary and sufficient for producing obesity-induced AHR (69). Besides, IL-17A is a potent neutrophil chemotactic agent, which explains the association between obesity-induced asthma and neutrophilia (70).

Obesity can also exacerbate allergic asthma. Indeed, HDM-induced airway inflammation and AHR in mice are worsened by high-fat diet-induced obesity. This effect is mediated by both ILC2s and ILC3s since obesity on its own increased both ILC types in the lungs and specifically depleting ILCs profoundly decreased HDM-induced asthma (71). How obesity stimulates ILC2s is not yet clear, but it may also involve NLRP3 inflammasome-derived IL-1β since this cytokine is upregulated in obesity (68) and was recently shown to promote the type-2 cytokine production of ILC2s (72). Other forms of non-allergic asthma are caused by environmental pollutants such as particulate matter (PM), diesel exhaust particles (DEP), ozone, and carbon nanotubes: the latter mimic the products of engineered nanomaterial degradation (73–75). A systemic review of *in vivo* and *in vitro* studies concluded that PM, DEP, and high ozone doses induce airway inflammation by regulating ILCs. They stimulate lung ILC2s to produce high levels of IL-5 and IL-13, which induces AHR (76). Besides, it is supported by correlations between peripheral ILC2 frequencies in humans and the PM_10_ and PM_2.5_ levels in the area they live in (75).

Interestingly, it has been suggested that the non-eosinophilia in non-allergic asthma is mediated by ILC1- and ILC3-induced activation of M1 macrophages whereas the eosinophilia in allergic asthma is generated by ILC2-mediated M2 macrophage activation (8). Thus, it is possible that ILCs drive the eosinophilia or neutrophilia in particular asthma phenotypes at least in part by shaping macrophage polarization. These findings together suggest that the different ILC types may play important roles in human asthma heterogeneity.

## Memory-like features of innate lymphoid cells

Most studies assessing the role of ILC in asthma have used acute models of asthma. However, since asthma is a chronic disorder whose symptoms persist after antigen clearance, it is critical to determine the mechanisms by which asthma endures (50, 77, 78). To address this, Christianson et al. developed a model of chronic asthma by intranasally treating mice with three different allergens (HDM, ragweed, and *Aspergillus*) twice a week for six weeks. They observed that airway inflammation and AHR persisted for six months after ceasing this treatment. They then noted that ILC depletion or IL-33 blockade significantly eliminated the airway inflammation, whereas T cell depletion had no effect. Thus, ILC2s, but not T cells, play a pivotal role in asthma persistence (50). Crucially, this study also suggested that ILCs might have immunological memory since they remained active long after the triggering antigen had disappeared.

The notion of ILC memory was supported by the detection of ILC2s that express CD45RO in human inflamed mucosal tissue (79). Since CD45RO is a widely used marker of memory T cells while CD45RA signifies naïve T cells (80, 81), it was suggested that CD45RO^+^ ILC2s could be memory-like ILCs. Indeed, it was observed that alarmins stimulation induces CD45RA^+^ ILC2s to differentiate into CD45RO^+^ ILC2s and that the latter cells produce more cytokines than CD45RA^+^ ILC2s (79). In addition, the latest research by Ham et al. memory-like CD45RO^+^ ILC3s increased in smoker asthmatics blood. CD45RO^+^ circulating ILC3s were associated with the asthmatic severity and counts of the peripheral blood neutrophils (9). Below, we discuss the current understanding of innate immune memory, memory-like ILCs, and the potential roles of ILC memory in asthma.

### Memory in innate immunity

**Figure 2:**
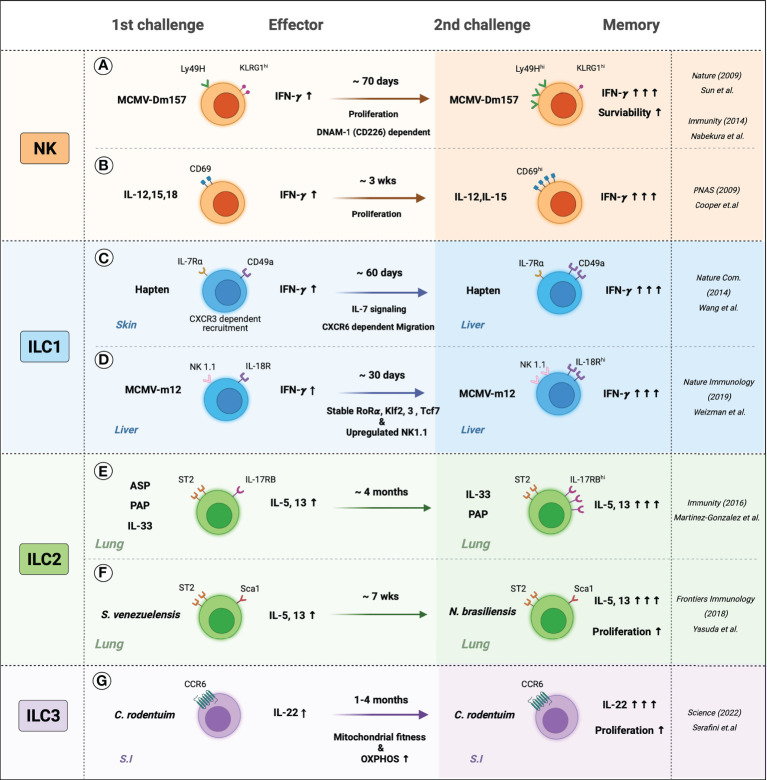
The memory-like features of ILCs. Generation and persistence of the memory ILCs are induced by the response to specific-antigen or cytokine signals. **(A)** Mice were infected with MCMV, Ly49H^+^ NK cells sensed MCMV glycoprotein m157 expressed more Ly49H and produced more IFN-γ and persists memory phenotypes, which may be involved by DNAM-1 (CD226) dependent. **(B)** *In-vitro*-primed CD69^+^ NK cells with IL-12, IL-15, and IL-18 displayed IFN-γ secretion when re-stimulated with IL-12 and IL-18. Primed NK cells produced more IFN-γ and showed increased CD69 expression. **(C)** Hapten-sensitized mice increased the numbers of IL-7Rα^+^ ILC1s and were recruited in the inflammatory site CXCR6-dependent manner. Hapten re-sensitization increased IL-7Rα^+^ ILC1s and IFN-γ. **(D)** Liver-resident IL-18R^+^ ILC1s are sensed MCMV m12, which leads to stable expression of the unique transcription factors such as Rorɑ, Klf2, 3, and Tcf7 and upregulated expression of NK 1.1. Also, MCMV-m12-induced memory ILC1s produced more. **(E)** Intranasal allergen and alarmin; ASP (Aspergillus), PAP (Papain), and IL-33 elevated ILC2s cytokines and maintained activated phenotypes. Re-challenging ILC2s with allergens or IL-33 induced higher production of IL-5 and IL-13. **(F)** *S. venezuelensis*-experienced mice have resistant to *N. brasiliensis* than naïve mice by generating more cytokine-producing memory ILC2s. **(G)** the small intestine (S.I) resident memory ILC3s produced more IL-22 and persisted for several months. *C. rodentium*-induced memory ILC3s experienced greater mitochondrial fitness and high OXPHOS rates.

The long-held dogma in the immunological field is that immunological memory is a hallmark of adaptive T and B cells: these cells display a slow response when first exposed to the specific antigen, but after the antigen clearance, they became long-lived memory cells that respond rapidly and strongly on subsequent exposures. By contrast, innate immune cells have been considered to respond to only the immediate and non-specific responses in the early onsets of diseases (82, 83). However, this traditional concept was challenged recently by the discovery of pattern recognition receptors (PRRs) that recognize specific microorganism components called pathogen-associated molecular patterns (PAMPs): this finding suggests that innate immune cells could have antigen specificity (82, 84). In addition, innate immune cells seem to remember previous stimuli and acquire long-term adaptations that enhance or lower responses after subsequent stimulation. For example, mice lacking T and B cells are protected from *Candida Albicans* reinfection by monocytes that recognize β-glucans in the fungal cell wall with receptors called a dectin-1 receptor, compared to naïve monocytes. Monocytes from previously infected mice demonstrate enhanced *in vivo* and *in vitro* cytokine production when challenged with *C. Albicans* or β-glucans (85–87). This phenomenon is designated as innate immune memory or trained immunity. Most studies on this phenomenon have focused on monocytes, monocyte-derived macrophages, and NK cells. However, innate memory in monocytes and macrophages has been well-reviewed in-depth recently (88, 89). Therefore, we will focus here on ILC memory and recap the scattered concepts of memory ILCs (**Figure 2**).

### Memory in innate lymphoid cells

#### Memory in NK cells

The first evidence of ILC memory was found in the contact hypersensitivity (CHS) model. While it was long thought that CHS is mediated by T cells, O’Leary et al. observed that different T and B cell-deficient (*Rag^-/-^*, SCID, and nu/nu) mice also demonstrate CHS when exposed to specific haptens. Moreover, CHS is larger on re-exposure to the hapten. This augmented response is specific to the hapten and is observed at least four weeks after the primary exposure. It was then found that the responses are mediated by NK cells in the liver: transfer of these cells into naive mice generated immunological memory, namely, augmented hapten-specific CHS responses (90).

Several mechanisms by which NK cells acquire a memory phenotype have been proposed. One involves pathogen recognition receptors (87). Ly49H is a type 2 C-type lectin-like membrane glycoprotein that belongs to the Ly49 receptor family, whose members interact with MHC and recognize MHC-bound peptides (91). Sun et al. showed that when mice are infected with murine cytomegalovirus (MCMV), NK cells demonstrated elevated expression of Ly49H and respond specifically to the MCMV glycoprotein m157. These cells persist for at least 70 days, degranulate more rapidly, and produce significantly more IFN-γ on reinfection. They protect naïve mice from MCMV when they are adoptively transferred (87, 92). Nabekura et al. then showed that the Ly49H-mediated memory of NK cells in the MCMV model may also involve the co-stimulatory molecule DNAM-1 (DNAX helper molecule-1, CD226): antibody blockade of DNAM-1, or DNAM-1-deficiency (*Cd226^−/−^*), associated with the impaired formation of MCMV-specific Ly49H^+^ NK cell memory. DNAM-1 was then found to be essential for the differentiation of effector and memory Ly49H^+^ NK cells (93).

Another proposed mechanism by which NK cells acquire a memory phenotype involves cytokines: several have been shown to induce NK memory without the need for specific antigen recognition. Thus, Cooper et al. showed that when NK cells are primed *in vitro* with IL-12, IL-15, and IL-18 and then transferred into *Rag1* ^-/-^ mice, the donor cells display augmented IFN-γ secretion when they are retrieved from the spleen and re-stimulated with IL-12 and IL-18. Moreover, upregulated IFN-γ secretion was detected more than three weeks after adoptive transfer and was maintained regardless of how many times the cells had proliferated (94).

Together, these studies point to the existence of NK memory. Further research elucidating the molecular mechanisms underlying NK cell memory is warranted since they could lead to novel NK cell-based vaccines and therapies.

#### Memory in ILC1s

ILC1 cells were often confused with NK cells in early studies because both secrete large amounts of IFN-γ. However, recent studies that excluded NK cells from ILC1 preparations show that ILC1 can also have memory. Thus, Wang et al. sensitized mice to hapten and divided the hepatic innate lymphocytes of naïve and sensitized mice into three types, namely, conventional NK cells (CD49a*^−^* IL-7Rα*^−^*), liver-resident NK cells (CD49a^+^ IL-7Rα*^−^*), and ILC1s (CD49a^+^ IL-7Rα^+^). The hapten sensitization increased ILC1 expression of IL-7Rα, and the absolute number of IL-7Rα^+^ ILC1s was doubled for at least 60 days. Re-sensitization produced augmented CHS responses. Adoptive transfer of the ILC1s but not the NK cell populations into naïve mice resulted in elevated CHS responses on hapten exposure. The molecular mechanism by which ILC1 memory arose was not determined; however, it involved ILC1 priming with hapten in the skin-draining lymph nodes, their exit into the blood, and their preferential recruitment *via* CXCR6 into the liver, where they then resided and maintained longevity through IL-7 signaling. On skin exposure to a hapten, the memory ILC1s migrated out of the liver and induced vigorous local CHS responses (95). Weizman et al. recently observed that ILC1s also gain memory of MCMV infection. Thus, they noted that MCMV infection induces liver-resident ILC1s to proliferate and express more IL-18 receptor (IL-18R). The elevated IL-18R expression maintained for at least 30 days after MCMV infection. On reinfection, the liver-resident IL-18R^+^ ILCs produced more IFN-γ, and the response was dependent on the MCMV m12 antigen (96).

Thus, ILC1 can also develop memory. It should be noted that the studies on these cells are limited to the liver and resemble antigen-specific NK cell memory. It is possible that these cells can also acquire memory in other tissues, including the lung, and that different mechanisms may be involved, however, further research is warranted.

#### Memory in ILC2s

Memory-like ILC2s in the lung have been well-reviewed by Martinez-Gonzalez and colleagues (97, 98). Thus, intranasal administration of naïve mice with allergens (papain, fungal protease, or recombinant IL-33) elevates cytokine production from lung ILC2s while elevating their numbers by 10 to 100-fold (19, 52, 55). These responses then decline, but some ILC2s that were generated during the early response persist long after the resolution of inflammation. For example, BrdU-labeled lung ILC2s were detected more than two months after IL-33 stimulation (19). Re-challenging the mice associated with significantly higher production of IL-5 and IL-13, including when *Rag^-/-^* mice are used (19).

Memory ILC2s in the lung were also observed through the establishment of memory immunity by infection with the nematode *Strongyloides venezuelensis* and the unrelated nematode *Nippostrongylus brasiliensis*. Both parasites enter the host body through the skin, travel to the lungs, and then migrate to the intestines. Both parasites also induce eosinophilic lung inflammation that associates with ILC2 accumulation (99). However, the parasites generate qualitatively different T_H_2 immune responses: thus, *N. brasiliensis* is expelled by T_H_2 cytokine-induced goblet-cell induction, epithelial-cell turnover, and smooth-muscle contraction, while *S. venezuelensis* is eliminated by T_H_2 cytokine-induced mastocytosis and antibody/FcRγ-dependent mucosal mast cell activation (100). Recently, Yasuda et al. showed that *S. venezuelensis*-experienced mice were significantly more resistant to *N. brasiliensis* than naïve mice, including when CD4^+^ T cells were depleted. This was due to the acquisition of highly responsive trained ILC2s in the lungs. These cells produced large amounts of IL-5 and IL-13 and induced early eosinophilia in the lung when encountering the second nematode. Reversing the sequence of nematode infection also induced memory ILC2 responses, which suggests that ILC2 memory is not pathogen-specific (100). The mechanisms by which lung ILC2s gain memory and are maintained remain poorly understood, therefore, further research is needed.

#### Memory in ILC3s

ILC3s and LTi cells are abundant in the gut, and their initial encounter with the gut microbiome modifies their relative distribution and cytokine production (101). Recently, Serafini et al. reported, for the first time, that ILC3s can also have memory properties. The murine model they used involved *Citrobacter rodentium*, which induces severe gastrointestinal inflammation. They noted that when previously infected mice were challenged with *C. rodentium*, intestinal ILC3s proliferated. These cells also generated more IL-22 than naive ILC3s after infection and controlled the infection better. In addition, these ILC3s persisted for several months. The development of trained ILC3s was also found when *Rag2^−/−^* mice were used, and when the pathogen was changed to *Listeria monocytogenes.* Thus, intestinal ILC3 memory is T-cell-independent and not limited to specific pathogens (20). These observations suggest that the gut microbiome could promote functional changes in intestinal ILC3s by generating long-term memory responses.

### Molecular changes that lead to memory of ILC

The studies described above show that the memory phenotype of NK cells can be mediated by the permanent upregulation of pathogen recognition receptors (87, 94, 102). Indeed, many studies on innate immune cells show that memory in these cells is associated with epigenetic and transcriptional changes (88, 89, 103). The epigenetic modifications regulate the expression of genes that control immune responses and include remodeling of chromatin structures, post-translational modifications of histones, DNA methylation, and the expression of non-coding RNAs. Interestingly, a comparison of MCMV-stimulated NK cells and CD8^+^ T cells showed that these cells acquire memory phenotypes *via* a similar epigenetic process (16). Moreover, chromatin accessibility analysis and transcriptional profiling show that memory NK cells differ from differentiated NK cells in terms of epigenetic/transcriptional signatures. In particular, the memory NK cell profile reveals a “poised” program that allows the cells to respond to their trigger more quickly (16). Similarly, Rasid et al. observed that the formation of memory NK cells involves histone modifications in the IFNG enhancer (Histone H3 lysine 4 Monomethylation (H3K4me1)) that are retained after activation signaling subsides. This modification permits the cells to transcribe IFN-γ faster when they are restimulated (15).

The studies on memory ILC1, ILC2, and ILC3s described above suggest that they gain their memory phenotype by similar mechanisms. Thus, the Martinez-Gonzalez group showed that the ILC2-mediated memory responses to allergens in the lung are induced by epithelial cell-derived IL-33, IL-25, and TSLP (rather than specific antigens). It may reflect permanent upregulation of the receptors for these cytokines: for example, naïve lung ILC2s express low IL-25R levels, whereas memory ILC2s display a sustained increase in IL-25R expression (19). Moreover, RNA-seq and ATAC-seq analyses of the MCMV-induced liver-resident IL-18R^+^ m12-specific memory ILC1s described by Weizman et al. showed that the memory ILC1s bore unique and stable transcriptional, epigenetic, phenotypic, and functional changes compared to naïve ILC1s. For example, NK1.1 was upregulated on the memory ILC1s: since the memory ILC1s displayed enhanced IFN-γ production when NK1.1 was stimulated, it is possible that m12-NK1.1 binding is responsible for the greater IFN-γ production of the memory ILC1s to MCMV. Alternatively, the upregulation of NK1.1 may reflect the reprogramming of the ILC1s that caused them to become memory cells, or it could be involved in the long-term maintenance of these cells (96).

ILCs may also gain memory by altering their metabolism. For example, the study by Serafini et al. on the *C. rodentium* infection-induced intestinal memory ILC3s observed that these cells exhibited high cellular oxygen consumption rates, which is indicative of aerobic glycolysis and greater mitochondrial fitness (20). Since such fitness is associated with innate immune signaling (104), it is possible that higher IL-22 production of the memory ILC3s is due in part to this metabolic change. The mechanisms by which metabolic changes induce ILC memory remain to be elucidated. One possibility is that the signaling cascades induced by the memory-inducing external stimuli generate metabolic products that act as signal transduction molecules, cofactors, and substrates that then modulate chromatin-modifying enzymes (14, 18). Another possibility is that metabolites modulate the transcriptional regulation of ILCs: numerous studies show that ILC-mediated immune responses are controlled by dietary components and metabolites (105). Moreover, metabolite and nutrient receptor analyses showed that ILC2s take up amino acids to maintain OXPHOS in a steady state (106). These observations together suggest that ILCs could modulate tissue-specific immune responses by acting as key sensors that integrate nutrient and metabolic stress. Given that metabolic changes alter the metabolite profile, which in turn can yield epigenetic modifications that drive memory ILC formation, it will be of interest to elucidate the metabolic controls on different ILC responses.

## Memory ILCs in asthma heterogeneity

The majority of asthmatics have allergic asthma, a disease caused by repeated allergen sensitization (107, 108). Animal models and studies on asthmatic patients show that along with T_H_2 cells, ILC2s play an essential role in allergic asthma pathogenesis (46). It may be particularly true for memory ILC2s for several reasons. First, memory ILC2s in papain-sensitized and challenged murine lungs produce as many type-2 cytokines as the memory T_H_2 in these tissues (19). Second, ILC2-derived IL-13 is essential for T_H_2 activation (60), and memory ILC2s produce more IL-13 than ILC2s that have encountered the triggering stimulus for the first time (19). Thus, memory ILC2s may contribute to allergic asthma by adding to the pathogenic type-2 cytokine load and enhancing allergen-specific T_H_2 responses.

The studies on ILC2 memory (see section above) suggest that epithelial-driven alarmins may be a trigger of ILC2 memory. There is evidence that this relatively unspecific trigger could be responsible for a well-known phenomenon in asthma, namely, that allergen-sensitive individuals often respond to unrelated stimuli (109). This is indicated by a study on late-onset allergic asthma, a severe form of asthma characterized by mixed eosinophil/neutrophil airway inflammation and respiratory fungal infection (110). The repeated intranasal challenge of fungi, such as *Aspergillus* and *A. alternate*, causes severe mixed eosinophilic/neutrophilic asthma. Notably, when these sensitized mice are re-challenged with the unrelated allergen papain, they developed significantly more airway inflammation than when naïve mice are challenged. Thus, memory ILC2s generated by the first stimuli can react to other allergens (19). The following evidence showed that this cross-sensitization may reflect IL-33-mediated ILC2 training. First, IL-33 expression in lung epithelial cells is induced by both papain and the fungal chitin/β-glucans. Second, the administration of IL-33 in fungus-experienced mice generates the same strong airway inflammation (111).

Such ILC2 memory training by epithelial-derived cytokines may also explain why virus infections exacerbate asthma. Such exacerbations are common in young children (112) and may reflect fundamental immune differences between children and adults and the fact that viruses also induce the airway epithelium to release cytokines that trigger memory ILC2s. Thus, an early study by Chang et al. showed that influenza infection of the adult murine lung induced AHR dependent on ILCs but not T cells (44). Saravia et al. then showed that when murine neonates were infected with a respiratory syncytial virus, IL-13-producing ILC2s were generated. This effect depended on epithelium-derived IL-33 production, and led to excessive mucus production and AHR. This study also showed that compared to adult mice, neonatal mice had more ILC2s and produced more IL-33 in their lungs in response to the virus (113). In addition, Steer et al. observed that when neonatal lungs were treated with IL-33, ILC2s were generated and persisted into adulthood, where they induced more intense responses to IL-33 administration. Notably, analyses of purified naïve and memory ILC2s from adults and neonates showed that naïve neonatal ILC2s resemble memory adult ILC2s in that they express high levels of the receptor for IL-25, another epithelial alarmin. By contrast, naïve adult ILC2 lack such expression. It means that neonatal ILC2s can respond immediately to IL-25, whereas adult ILC2s only gain that capability when they become memory cells (114). Thus, memory ILC2s generated by epithelial alarmins may be a major mechanism by which viruses exacerbate asthma.

Whether memory ILCs also participate in non-allergic asthma, including pollutant-, obesity-, and ozone-related asthma, is not yet clear. However, there are hints that they do. Thus, individual memory T cells tend to produce multiple cytokines (115), which suggests that memory ILCs could also bear this multi-functionality. It is possible this occurs in non-allergic asthma since this endotype often exhibits a mixed T_H_2 and T_H_17 phenotype. In other words, non-allergic asthma may be characterized by memory ILCs that produce both type 2 and type 17 cytokines. It is supported by studies on obesity-induced asthma as follows: (i) obesity on its own increases both ILC2 and ILC3 responses in the lungs (71); (ii) obesity-induced AHR requires IL-17-producing ILC3s (69); and (iii) when obese mice are exposed to ozone [which on its own induces airway inflammation in an IL-33-ILC2-dependent manner (116)], their AHR is amplified *via* a mechanism mediated by IL-13-producing ILC2s (117). Thus, obesity-induced asthma may associate with both ILC2 and ILC3 responses. It should be noted that NLRP3 inflammasome-derived IL-1β plays an important role in the obesity model: this cytokine is upregulated in obesity (68), and blockade of IL-1β abolishes obesity-induced ILC3-mediated AHR (69). It may participate in the mixed T_H_2 and T_H_17 cytokine phenotype in obesity since it promotes the T_H_17 cytokine production of ILC3s (69), the T_H_2 cytokine production of ILC2s (71), and primes ILC2s to become more responsive to IL-33 (117). However, since ILCs are highly plastic and IL-1β controls ILC2 plasticity (72, 118), it is also possible that the mixed cytokine phenotype in obesity may reflect ILC plasticity rather than multifunctional memory ILCs.

Although recent studies about memory ILC1s were mainly focused on the liver, it may have a potential that memory ILC1s can also participate in non-allergic asthma pathogenesis. Since Kim et al. (8) previously reported the increase of ILC1s in asthmatic patients, activated memory ILC1s may exacerbate asthma phenotype by producing higher amounts of T_H_1 cytokines such as IFN-γ. Environmental pollutants such as DEP, PM, and ozone or chronic inflammation cause epithelial tissue damage and may produce cytokines such as IL-12, IL-15, and IL-18 (73–75). However, how these air pollutants contribute to the ILC1 memory formation that may cause non-allergic asthma needs further research.

Bacterial and viral infection-induced asthma exacerbations and obesity-induced asthma are associated with resistance to steroids, the main treatment for asthma (6, 119, 120). Several studies suggest that this resistance is mediated by ILC2s (121–123). It is possible that memory ILCs participate in steroid resistance since Van der Ploeg et al. showed that patients with steroid-resistant chronic rhinosinusitis or asthma have higher CD45RO^+^ ILC2s in their mucosal tissue and blood than healthy controls. Especially, epithelial alarmins such as IL-33 and TSLP induced resting CD45RA^+^ ILC2s to convert into CD45RO^+^ ILC2s. Besides, CD45RO expressing ILC2s showed more potential to produce type 2 cytokines (79). Moreover, the possibility of memory ILCs is further supported by Ham et al., which showed that the CD45RO expressing ILC3s are elevated in smoking asthma patients and that the CD45RO^+^ ILC3 frequencies correlated positively with disease severity (9).

**Figure 3:**
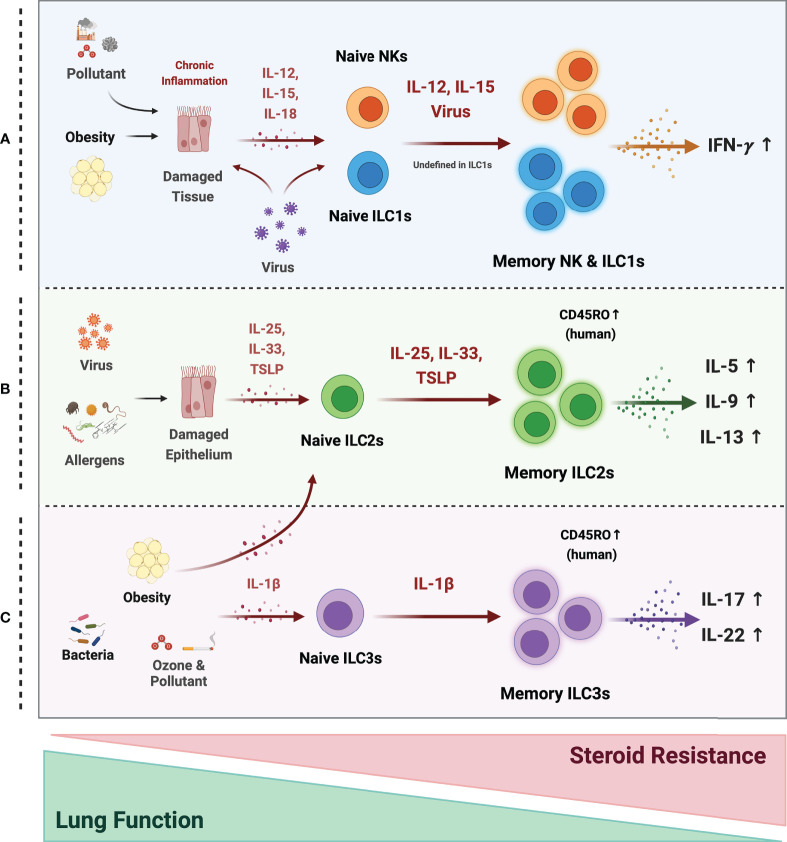
Possible mechanism of memory ILCs in asthma pathogenesis. Memory properties of ILCs may participate in the pathogenesis of heterogeneous asthma associated with excessive cytokines. **(A)** In obesity, air pollutant-associated asthma, and viral infection, chronic inflammatory environments, induce tissue damage and release tissue-derived cytokines (IL-12, IL-15, IL-18). NK cells may acquire memory function and secrete more IFN-γ in response to epithelial-derived stimuli or directly by the virus. The roles of ILC1s are still ambiguous, but they also respond to tissue-derived cytokines and secrete large amounts of IFN-γ under chronic inflammation. **(B)** Memory ILC2s may contribute to most asthma endotypes induced by a viral infection and repetitive allergen sensitization. Repeated allergens exposure increased alarmin expression on the epithelium. Memory ILC2s from the activated Naïve ILC2s by alarmins, produce excessive type 2 cytokine production. **(C)** In non-allergic asthma caused by bacterial infection, environmental stimuli (ozone and pollutant), and obesity, the ILC2s and ILC3s may acquire memory traits in response to IL-1β. They produce more IL-17A and 22 at the ILC3s and more type 2 cytokines at the ILC2s. ILC2 and ILC3 populations expressing the memory T cell marker CD45RO are also reported in asthmatics. These cells are more active and associated with decreased lung function and increased steroid resistance.

Thus, memory ILCs may be involved in asthma pathogenesis and may help explain why asthma associates with diverse antigens and presents as different endotypes (allergic and non-allergic) and phenotypes (e.g., infection-induced asthma exacerbations in children, obesity-induced asthma, and steroid-resistant asthma) (**Figure 3**). Further research is needed to unravel the precise mechanisms by which memory ILCs are induced and act in asthma since it could provide new perspectives regarding asthma treatment strategies.

## Concluding remarks

This review delineates the biology of ILCs, their role in asthma pathogenesis, and the studies that suggest memory ILCs contribute to asthma. Novice and trained ILCs play crucial roles in asthma pathogenesis. Since (i) they can respond to various stimuli, including pathogen-derived ‘PAMP’ and tissue-derived ‘DAMP’ signals, (ii) they are active effectors of asthma pathogenesis due to their production of enormous amounts of cytokines. And (iii) they demonstrate memory, namely, longevity and robust and rapid responses to various allergens or alarmins on re-exposure. This memory phenotype is associated with reprogramming the ILC genetic code and metabolic machinery. While much more work is needed to decode the mechanisms that generate ILC memory, research in this area may likely help shed light on vexing unresolved issues in asthma, including why asthma is so heterogeneous and why some forms exhibit steroid resistance.

## Author contributions

Conceptualization: HK. Investigation: JH, ML, DK. Writing - original draft: JH, ML, DK, HK. Writing - review & editing: JH, ML, DK, HK. All authors contributed to the article and approved the submitted version.

## Funding

This work was supported by the National Research Foundation of Korea (2022R1A2C3007730, 2021M3A9I2080493 and SRC2017R1A5A1014560) and the Cooperative Research Program of Basic Medical Science and Clinical Science from Seoul National University College of Medicine (800-2021288).

## Acknowledgments

Cartoons in **Figures 1**–**3** were created with BioRender.com.

## Conflict of interest

The authors declare that the research was conducted in the absence of any commercial or financial relationships that could be construed as a potential conflict of interest.

## Publisher’s note

All claims expressed in this article are solely those of the authors and do not necessarily represent those of their affiliated organizations, or those of the publisher, the editors and the reviewers. Any product that may be evaluated in this article, or claim that may be made by its manufacturer, is not guaranteed or endorsed by the publisher.
